# Implement a knowledge‐based automated dose volume histogram prediction module in Pinnacle^3^ treatment planning system for plan quality assurance and guidance

**DOI:** 10.1002/acm2.12689

**Published:** 2019-07-25

**Authors:** Hao Xu, Jiayu Lu, Jiazhou Wang, Jiawei Fan, Weigang Hu

**Affiliations:** ^1^ Department of Radiation Oncology Fudan University Shanghai Cancer Center Shanghai China; ^2^ Department of Oncology Shanghai Medical College, Fudan University Shanghai China

**Keywords:** Knowledge‐based, KDE, DVH, QA, Pinnacle^3^

## Abstract

**Purpose:**

This work aims to develop a knowledge‐based automated dose volume histogram (DVH) prediction module that serves as a plan quality evaluation tool and treatment planning guidance in commercial Pinnacle^3^ treatment planning system (Philips Radiation Oncology Systems, Fitchburg, WI, USA).

**Methods:**

The knowledge‐based automated DVH prediction module was developed with kernel density estimation (KDE) method and applied for Pinnacle^3^ treatment planning system. Treatment plan data from 20 esophageal cancer cases were used for creating a module to predict DVHs. Twenty additional esophageal clinical plans were evaluated on the developed module. Predicted DVHs were compared with manual ones. Differences between the predicted and achieved DVHs were analyzed.

**Results:**

The plan evaluation module was successfully implemented in Pinnacle^3^ treatment planning system. Strong linear correlations were found between predicted and achieved DVH for organs at risk. Suboptimal treatment plan quality could be improved according to the predicted DVHs by the module.

**Conclusion:**

The knowledge‐based automated DVH prediction module implemented in Pinnacle^3^ could be used to efficiently evaluate the treatment plan quality and as guidance for further plan optimization.

## 1 INTRODUCTION

Intensity‐modulated radiation therapy (IMRT) is a popular clinical treatment modality used worldwide. Compared to conventional beams of uniform intensity, intensities of radiation beams are modulated in IMRT to deliver a nonuniform dose distribution to the tumor target. A desired target dose conformity and sufficient sparing of critical structures could be achieved through IMRT planning.^1^, ^2^, ^3^ An ideal IMRT plan could require a lot of trial‐and‐error process and was time‐consuming. The efficiency of making an IMRT plan and the quality of this plan depend on the clinical experience of the dosimetrist.^3^, ^4^, ^5^

The population of patients treated for esophageal cancer is increasing in our center. Esophageal plan quality has been investigated for different treatment modalities.^6^ Compared to three‐dimensional conformal radiotherapy (3DCRT), IMRT plans generally show better target dose coverages and lower mean doses to organs at risk (OARs). Also, high‐quality plans are desired to achieve optimal treatment outcomes.^7^ Therefore, a pretreatment plan quality assurance (QA) tool is crucial for assuring treatment plans.

Knowledge‐based dose volume histogram (DVH) prediction has shown good results for head and neck, pancreas and prostate treatment planning.^8^, ^9^, ^10^, ^11^, ^12^ The plan variability can be reduced through knowledge‐based planning.^13^, ^14^, ^15^, ^16^ Although there is no definitive method to evaluate a radiotherapy treatment plan, the feasibility of using knowledge‐based DVH prediction for QA of head and neck plan has been demonstrated.^17^ As a commercial knowledge‐based optimization engine, RapidPlan™ in the Eclipse treatment planning system (Varian Medical Systems, Palo Alto, USA) showed its potential to serve as an accurate plan QA tool. Strong correlations had been found between RapidPlan™ predicted and manually achieved mean doses to OARs. The predicted OAR sparing was validated by replanning the patient with the RapidPlan™ module. Suboptimal IMRT plan quality can be improved. Besides the knowledge‐based RapidPlan™ module in the Eclipse, Pinnacle^3^ treatment planning system (Philips Radiation Oncology Systems, Fitchburg, WI, USA) also provides an Auto‐Planning module using plan‐simulating for automated plan optimization.^15^, ^18^ However, it cannot predict OAR DVH. The dilemma on balancing plan efficiency and plan quality is popular in developing country with heavy patient load but limited dosimetrists. Therefore, a quick method to verify plan quality or guide the treatment planning is crucial in clinic.

We have proposed a new knowledge‐based auto‐planning solution for IMRT treatment planning.^19^ It used two parameters kernel density estimation (KDE) algorithm to build the DVH prediction model. The model feasibility in breast cancer has been validated with treatment plan quality and consistency. In this study, a different KDE method was developed and applied for esophageal cancer and integrated into the commercial Pinnacle^3^ treatment planning system to create a knowledge‐based automated DVH prediction module, which can be used for treatment planning QA or guidance.

## 2 METHODS AND MATERIALS

### 2.1 Overview of the knowledge‐based plan QA module workflow

**Figure 1:**
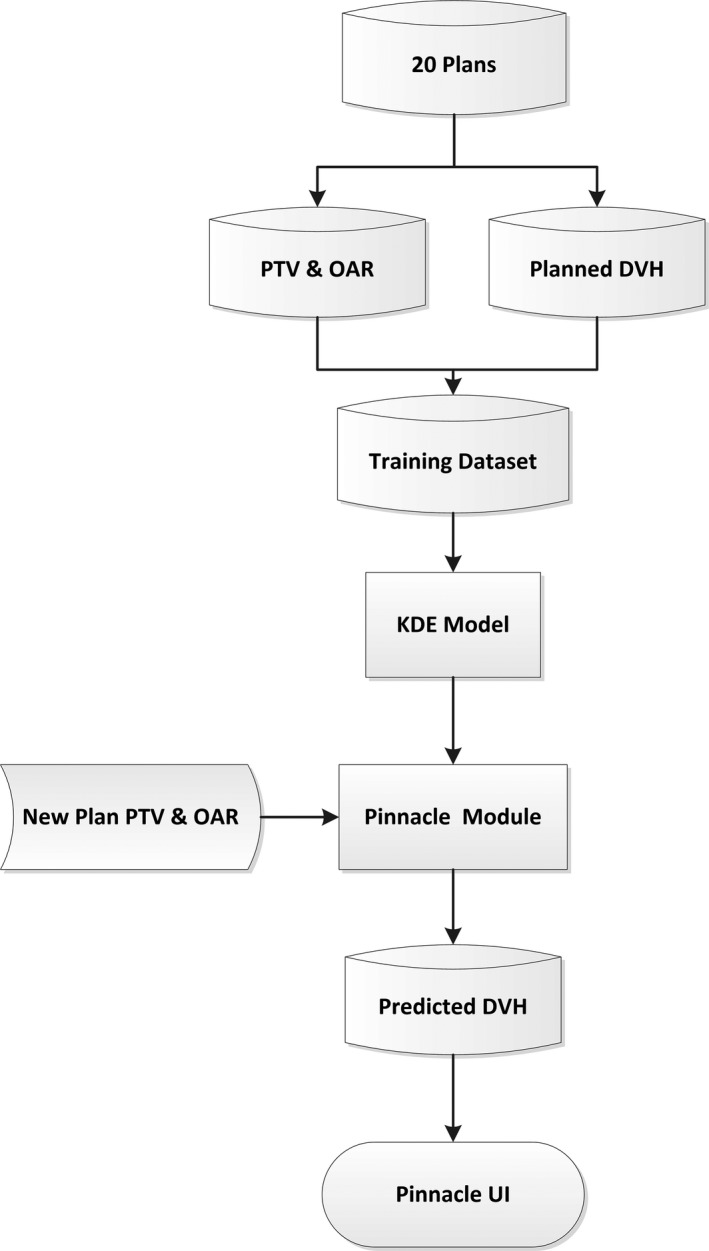
Schematic view of knowledge‐based plan quality assurance module workflow.

A schematic view of the plan QA module in Pinnacle^3^ is showed in Fig. 1. Twenty IMRT cases with the same prescription dose for esophageal cancer were planned by dosimetrists who had more than 10 years work experience in our institution. These manually optimized plans were clinically acceptable with high quality. DVH data, voxels of planning target volume (PTV) and OARs extracted from these plans were used as training data. A KDE model was trained with these data and integrated into the Pinnacle^3^ treatment planning system as a plan evaluation module. For new patient with PTV and OAR delineation, corresponded DVH prediction would be generated from this module. Predicted DVH curves can be shown on Pinnacle^3^ user interface (UI), which could be used as a benchmark to evaluate treatment plan quality or as guidance for further treatment optimization.

### 2.2 KDE‐based DVH prediction

Dose distribution, voxels of PTV and OARs extracted from the training data were trained to get DVH prediction. DVH can be determined by a cumulative probability distribution of dose x that is lower or equal to a given dose D as Eq. (1).

(1) $$\text{DVH}\left(\text{D}\right)=1-\int_{0}^{\text{D}}{\text{p}}_{\text{D}}\left(\text{x}\right)\text{dx}$$

The dose probability density function p_D_(x) was predicted by marginalizing the conditional probability density p(x|t) estimated from training data over probability density p*(t) from input plan data, where t was the signed minimal distance between dose point in OAR voxel and PTV boundary. When OAR overlapped PTV, signed distance of the point was negative inside PTV. For each input plan data, p_D_(x) can be estimated by Eq. (2).

(2) $${\text{p}}_{\text{D}}\left(\text{x}\right)=\int_{\text{t}}\text{p}\left(\text{x}|\text{t}\right){\text{p}}^{*}\left(\text{t}\right)\text{dt}$$

From KDE method which was introduced by Skarpman,^20^ to generate a continuous probability density function p_D_(x), Gaussian kernel was applied to the training data. The width of the Gaussian kernel in this KDE model was calculated by minimizing mean integrated squared error.^21^

### 2.3 KDE model validation

The KDE model was validated by 10 additional high‐quality treatment plans. Plan evaluation metrics were summarized in Table 1. DVH cut‐points (Vd) represented percent volume coverage of OAR that received dose higher than d (Gy). By calculating Eq. (3), performance of the model was evaluated with root mean square error (RMSE) by comparing the difference between predicted and achieved DVH metrics from corresponding validation data.

(3) $$\text{RMSE}=\sqrt{\frac{\sum \left(\text{Predicted}-\text{Achieved}\right)}{\text{Nplans}}}$$

**Table 1:** Evaluation metrics for esophageal IMRT plan

OAR	DVH
Heart	Mean (Gy)
	V_30_ (%)
Lung	Mean (Gy)
	V_20_ (%)
	V_5_ (%)
Lung‐PTV	Mean (Gy)
	V_20_ (%)
	V_5_ (%)
SC	Max (Gy)

Abbreviations: DVH, dose volume histogram; IMRT, intensity‐modulated radiation therapy; OAR, organs at risk; PTV, planning target volume.

### 2.4 Plan QA module implementation and user interface integration in Pinnacle^3^

Realization of plan QA function in Pinnacle^3^ treatment planning system has three parts: plan data access, data processing, and results feedback. Plan data from treatment planning system was imported through an interface developed in Java programming language and data processing was also performed in Java. The plan QA model was developed in R programming language. The predicted DVH from the model was embedded in the plan evaluation module of treatment planning system using Pinnacle^3^’s scripts.

### 2.5 Application and evaluation in clinical implementation

The developed QA module in Pinnacle^3^ was applied for 20 new esophageal IMRT plans with the same prescription dose. Plan quality evaluation was made with respect to the DVH metrics in Table 1. The discrepancy between predicted and achieved DVH metrics was analyzed statistically.

### 2.6 Statistical analysis

Clinical IMRT plans of esophageal cancer were evaluated between the manual DVHs and predicted DVHs from the QA module. Mean values and RMSE of data were used for statistical analysis. The statistics were calculated using SPSS (v13.0, IBM corp., New York, NY, USA). Regression analysis used R‐squared (R^2^) as the coefficient of determination. The coefficient of determination of the regression model would be close to 1 for a good fit.

## 3 RESULTS

### 3.1 KDE model validation

Performance of the KDE model for esophageal cancer plan is shown in Table 2. The mean values of the differences between predicted and achieved OAR metrics from validation plans were also summarized in the table. A 3.4% deviation of V_5_ index of lung‐PTV was found on average.

**Table 2:** Model validation

	Predicted‐to‐achieved value difference		
OAR		Mean	RMSE
Heart	Mean (Gy)	0.64	2.06
	V_30_ (%)	0.5	4.4
Lung	Mean (Gy)	0.2	1.32
	V_20_ (%)	0.9	4.9
	V_5_ (%)	1.7	6.7
Lung‐PTV	Mean (Gy)	0.34	1.1
	V_20_ (%)	1.1	4.4
	V_5_ (%)	3.4	6.3
SC	Max (Gy)	1.04	1.1

Abbreviations: OAR, organs at risk; PTV, planning target volume; RMSE, root mean square error.

### 3.2 Pinnacle^3^ user interface

**Figure 2:**
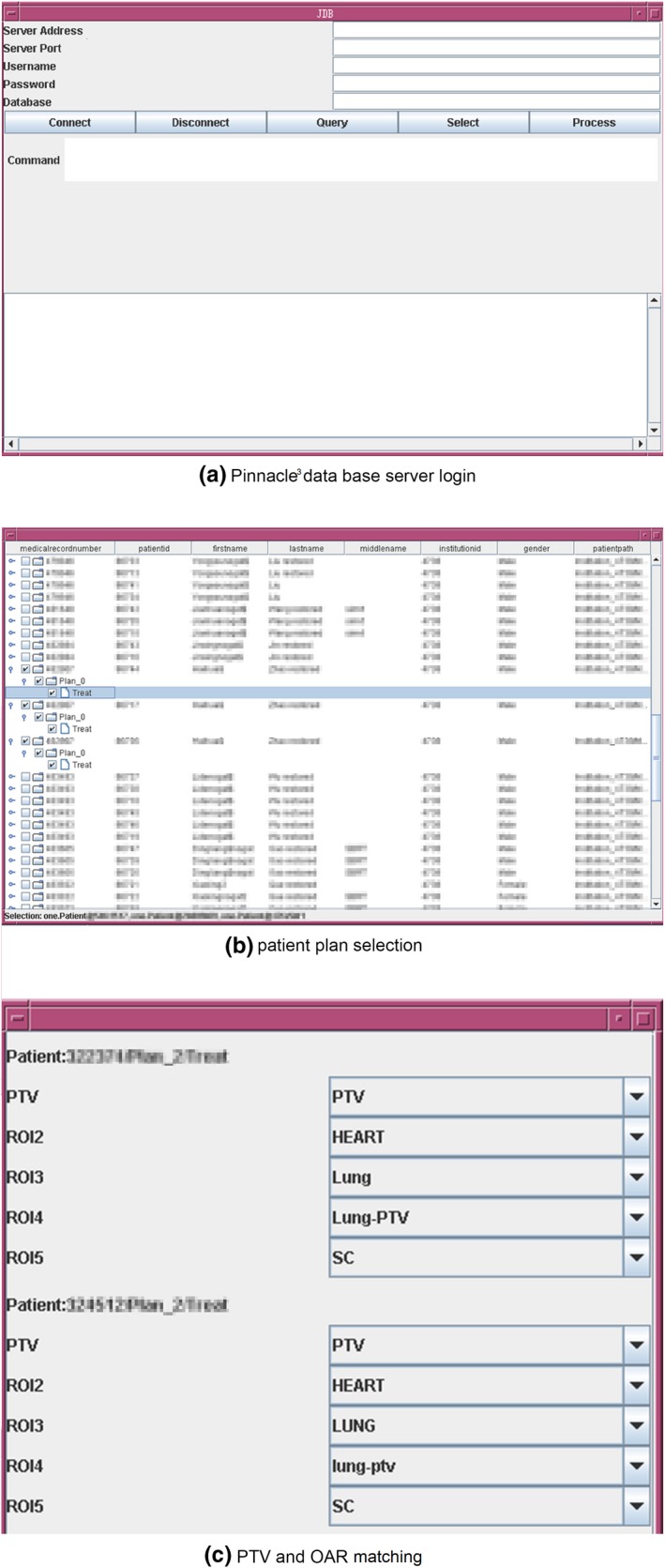
(a) Pinnacle^3^ data base server login; (b) patient plan selection; (c) planning target volume and organs at risk matching.

The developed Pinnacle^3^ UI for plan QA module has the following three parts: data base server login [Fig. 2(a)]; patient plan selection [Fig. 2(b)]; PTV and OAR matching [Fig. 2(c)]. In this study, 20 patients were selected using this UI for evaluation.

**Figure 3:**
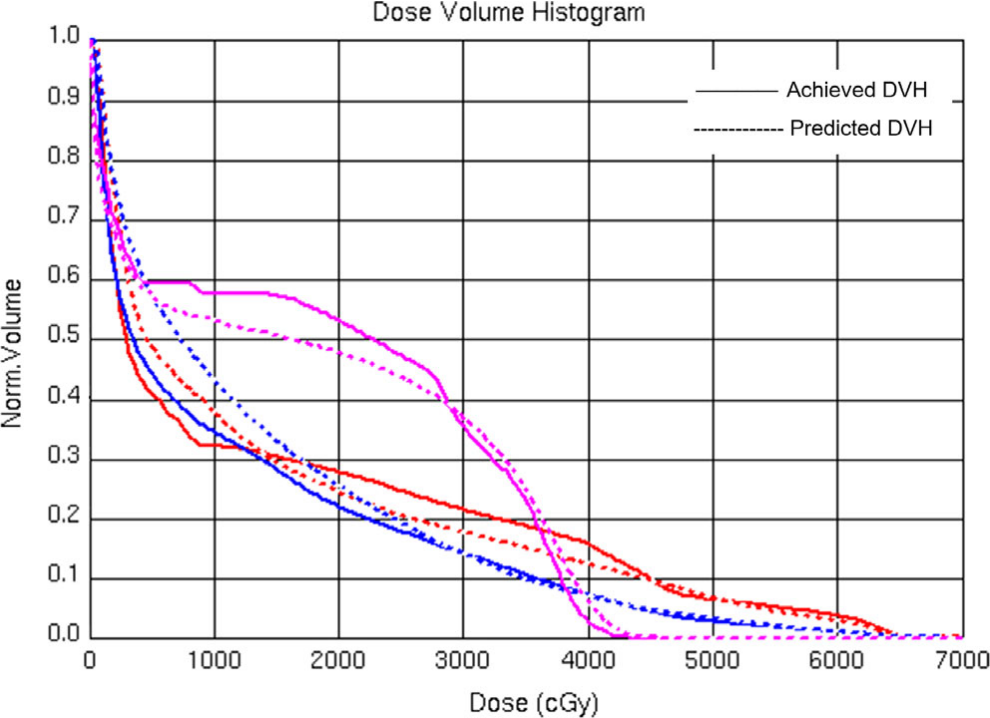
Plan quality assurance user interface in Pinnacle^3^.

Predicted DVH for plan QA was successfully integrated in the DVH display module in Pinnacle^3^ using the script. An example of plan evaluation UI in Pinnacle^3^ is shown in Fig. 3. The dashed line represents the predicted DVH and the solid line is the achieved DVH for the same OAR in treatment plan.

### 3.3 Clinical implementation

The dose comparison between predicted and manual DVHs was performed on 20 clinical plans. The OAR DVH comparison is shown in Table 3. A 1.2% deviation of V_20_ index and 2.5% deviation of V_5_ index of lung‐PTV were found on average.

**Table 3:** OAR DVH metrics comparison

	Predicted‐to‐achieved value difference		
OAR		Mean	RMSE
Heart	Mean (Gy)	−1.99	2.78
	V_30_ (%)	0.12	7.6
Lung	Mean (Gy)	−0.56	1.16
	V_20_ (%)	−1.2	4.0
	V_5_ (%)	−1.5	5.2
Lung‐PTV	Mean (Gy)	−0.59	1.25
	V_20_ (%)	−1.2	4.3
	V_5_ (%)	−2.5	5.8
SC	Max (Gy)	1.27	1.25

Abbreviations: DVH, dose volume histogram; OAR, organs at risk; PTV, planning target volume; RMSE, root mean square error.

**Figure 4:**
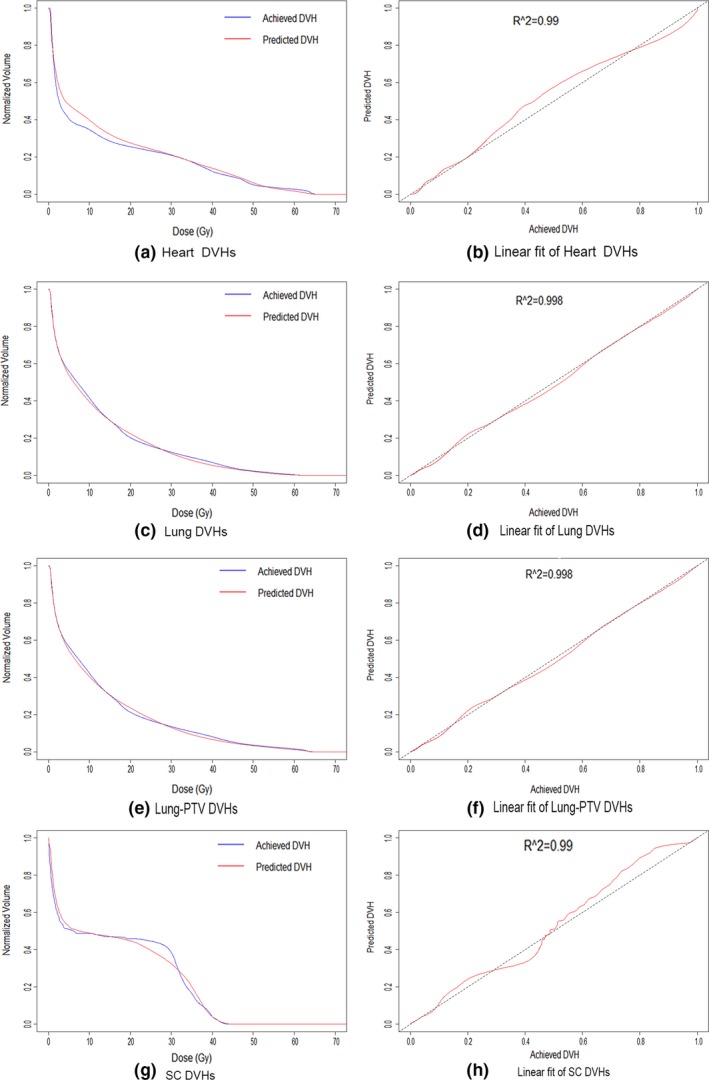
Achieved and predicted DVHs of different OARs and corresponding linear fits: (a) Heart DVHs; (b) Linear fit of Heart DVHs; (c) Lung DVHs; (d) Linear fit of Lung DVHs; (e) Lung‐PTV DVHs; (f) Linear fit of Lung‐PTV DVHs; (g) SC DVHs; (h) Linear fit of SC DVHs. The blue line is the original DVH and the red one is the generated DVH prediction. The predicted DVH data points were plotted against the achieved DVH data points for multiple OARs in the figure. The dashed line represents a linear fit with slope of 1. R^2^ values show the goodness of fit.

**Figure 5:**
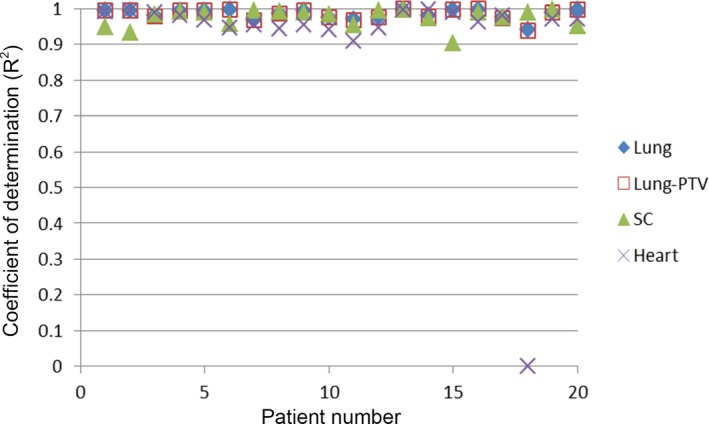
The coefficient of determination (R^2^) of dose volume histogram linear fit for all evaluated plans.

Figure 4 shows the achieved and predicted DVH curves for each OAR as a plan evaluation example using the model. The coefficient of determination (R^2^) of DVH linear fit for all plans evaluated are shown in Fig. 5. All linear fits had slopes toward 1 except an outlier of heart DVH linear regression. Lung and lung‐PTV both showed a strong linear correlation between predicted and achieved DVH data points with R^2^ values close to 1 (0.984 ± 0.015), which indicated that the predicted DVH curve nearly overlapped the achieved DVH curve. For spinal cord, the linear fit R^2^ values ranged from 0.904 to 0.996 (0.974 ± 0.025). The heart had R^2^ values ranging from 0.0078 to 0.997 (0.919 ± 0.216).

### 3.4 Predicted DVH‐guided treatment planning

**Figure 6:**
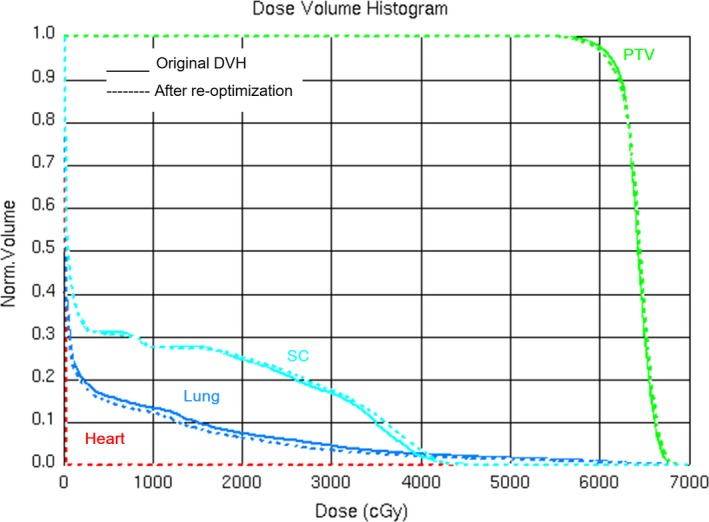
Results of re‐optimized plan. The solid lines represent original dose volume histogram (DVHs). The dashed lines were the re‐optimized DVHs.

To test the feasibility of treatment planning guidance using the predicted DVH, one of the 20 patients (patient number 18) from clinical test group with a slight lower predicted lung dose was selected. For this patient, the lung mean dose, V_5_, V_20_ in the manual plan was 1.0 Gy, 2.3%, 2.1% higher than the predicted ones, respectively. With the guidance of predicted DVH, a re‐optimization was performed. The final plan with re‐optimization is shown in Fig. 6. This indicated the feasibility of using predicted DVH as a treatment planning guidance.

## 4 DISCUSSION

The scope of this study was to apply a knowledge‐based automated DVH prediction module that could be integrated in Pinnacle^3^ UI for plan QA and guidance. To the best of our knowledge, this is the first time to develop the KDE‐based DVH prediction module in Pinnacle^3^. One of the 20 clinical esophageal IMRT plans was randomly selected out for re‐optimization under the guidance of predicted OAR DVHs. It was available to achieve an optimized result. We believe that this tool would be helpful for both plan QA and further DVH‐guided plan optimization or re‐optimization.

KDE method has shown good DVH prediction for Gamma knife radiosurgery of acoustic schwannoma and non‐small cell lung cancer treated with stereotactic body radiation therapy.^20^ The knowledge‐based module we developed with KDE for Pinnacle^3^ treatment planning system could be used to benchmark the quality of esophageal cancer plans and also to guide auto planning. With a training dataset consisting of clinical high‐quality plans created from expert dosimetrists, the DVH predictions would result in the best possible DVHs that could be expected in clinic. Automated individual plan QA would be possible by the developed module in Pinnacle^3^ as Rapidplan™ in Eclipse.^17^ There could be other better models for DVH prediction of esophageal IMRT plan, which needs to be investigated in the future.

A quantitative analysis of dosimetric accuracy of the knowledge‐based model was performed. For all manually optimized plans, strong linear correlations were found between predicted and achieved OAR DVHs. The achieved OAR mean doses were close to the predicted OAR mean doses. The consistency of good match between predicted and achieved OAR DVHs and mean OAR doses demonstrated that the developed plan QA model could evaluate plan quality effectively. The plans evaluated showed a consistently good level of OAR sparing. Higher maximum dose prediction of spinal cord could be a result of small number of samples in high dose region of DVH which would degrade the accuracy of KDE. Similarly, the OAR receiving low dose would result in insufficient sampling for low dose region estimation of DVH. Considering the geometric relationships between OARs and PTV in the training dataset, trade‐off scenarios among different OARs sparing would introduce different patterns of OAR DVH prediction with KDE. The model was trained with clinical IMRT plans for upper, medium, and lower third of the esophagus to build a generalized model for OAR DVH prediction. Therefore, any case of esophageal cancer IMRT plan could be available for QA with this model. Similar results were found in DVH estimation by RapidPlan™ using broad‐scope knowledge‐based model.^22^

Different planning techniques or field settings were not considered in this model. The model was unable to provide any evaluation results for triggering re‐optimization. That will be studied in the future. Broad knowledge‐based libraries could be built for different cancers in the future to generate specific plan QA models as a part of auto‐planning module in Pinnacle^3^. The planning knowledge from large centers would benefit relatively small centers to improve treatment plan quality and clinical outcome.

## 5 CONCLUSIONS

Implementation of a knowledge‐based automated DVH prediction module in Pinnacle^3^ has been successfully realized. The developed UI enables comparison between achieved and predicted DVHs. The feasibility of automated esophageal IMRT plan QA using the module has been demonstrated. Plan consistency and quality can be improved under the guidance of the module prediction.

## CONFLICT OF INTEREST

No conflict of interest.
